# Supplementary material for: A multichannel electrophysiological approach to noninvasively and precisely record human spinal cord activity
Source: PLoS Biol. 2024 Oct 31;22(10):e3002828. doi: 10.1371/journal.pbio.3002828 (PMC11527246; doi:10.1371/journal.pbio.3002828)
Supplement: S1 Text — This file contains descriptions regarding (i) analysis differences between preregistration and manuscript; (ii) results for mixed nerve stimulation from Experiment 2; (iii) results for the later spinal SEP components from Experiment 2; (iv) results from the sensory nerve stimulation in Experiment 2; (v) results from the analysis on changes in response amplitude across the processing hierarchy; and (vi) the choice of reference electrode. (PDF) [file pbio.3002828.s001.pdf]

Supplementary Text

A multi-channel electrophysiology approach to non-invasively and precisely record human spinal cord activity

Birgit Nierula, Tilman Stephani, Emma Bailey, Merve Kaptan, Lisa-Marie Pohle, Ulrike Horn, André Mouraux, Burkhard Maess, Arno Villringer, Gabriel Curio, Vadim V. Nikulin, Falk Eippert

Analysis differences between preregistration and manuscript

In this section, we list differences between the analyses proposed in the pre-registrations on OSF and the analyses we finally performed, as well as the reasons underlying these discrepancies.

Experiment 1: The preregistration stated that we aimed to also present SEPs at the channel with the strongest deflection. However, in the course of analyzing the data, we realized how well CCA was working on spinal data and decided that adding the time-course of the electrode with the strongest deflection would not bring additional value to the analysis, since CCA automatically incorporates the contribution of each channel to the SEP.

Experiment 1: The preregistration stated that we intended to investigate the relation between SEP amplitudes recorded at different levels of the somatosensory processing hierarchy. However, since we already show in Experiment 2 that there is mostly no such relation within one stimulation type, this analysis would not be very informative and we thus did not include it. Instead, we report a more informative analysis based on the data of Experiment 2, which allowed us to include different stimulation conditions (i.e., mixed, single-digit and double-digit stimulation)

Experiment 2: The preregistration stated that we wanted to include brainstem and Erb's point potentials in our analysis. However, due to low SNR we removed them from the results.

Experiment 2: The preregistration stated that we aimed to control for the difference in individual SEP latencies by taking the distance between the location of the recording and stimulation electrode into account. This was not necessary, because we used individual peak amplitudes and latencies in the present analysis.

Experiment 2: The preregistration stated that we intended to investigate the attenuation effect at the brainstem level (N14 and N30) as well. However, since the low SNR in the single-digit stimulation conditions did not allow for observing clear SEPs at the brainstem level, we were not able not to perform this analysis.

Experiment 2: The preregistration stated that for testing attenuation effects, we aimed to test the summed single-digit SEP-amplitudes against the double-digit amplitudes with a paired t-test. Since in the literature it is however more typical to calculate individual interaction ratios, we followed this approach and tested them against zero (Hsieh et al., 1995; Severens et al., 2010). However, for robustness we also performed paired t-tests and observed that this did not change the statistical decision (i.e., significant and non-significant comparisons remained in both analysis).

Mixed nerve results from Experiment 2

We also aimed to replicate the main results from Experiment 1 (N=36) using the data from the mixed nerve conditions in Experiment 2 (N=24) and list these results in S1 Table.

Later spinal SEP components

We aimed to replicate the late potentials observed in Experiment 1 with the data from the mixed nerve conditions in Experiment 2, using an identical approach. The following responses were identified via cluster-based permutation testing (after the early potentials, which are ignored here): i) in the hand mixed condition, we identified a cervical cluster directly after the N13 component between 19 ms and 24 ms (p_{mcc} = 0.012; channels: S3, S6, S7, S9, S11, S14, S18) that has higher

activity during stimulation than during rest and ii) in the foot-mixed condition, we identified a positive cluster directly after the N22 component between 29 ms and 35 ms (p_{mcc} = 0.004; channels: S22, S23, S26, L1, S28, S30, S32). This replicated the main results observed in Experiment 1, with the exception of the late potential displayed in S1 Fig.

Sensory nerve stimulation results

Detailed results from the sensory nerve stimulation conditions in Experiment 2 are listed in S2 Table. Comparisons with mixed nerve stimulation revealed that peaks following double finger stimulation (fingers1&2) occurred 3.91 ms (peripheral), 4.30 ms (spinal), and 3.90 ms (cortical) later than those following hand-mixed stimulation and were 76% (peripheral), 66% (spinal), and 59% (cortical) smaller in amplitude. Potentials following double toe stimulation (toes1&2) occurred 6.05 ms (peripheral), 7.63 ms (spinal), and 8.39 ms (cortical) later compared to mixed nerve stimulation and were 88% (peripheral), 63% (spinal), and 43% (cortical) smaller in amplitude. Statistical comparisons of these differences are listed in S3 Table.

Shared response properties across the somatosensory hierarchy

We here provide more extensive details regarding the investigation on whether response properties are shared across the somatosensory hierarchy.

Across the hand stimulation conditions, cortical SEP amplitudes were predicted by spinal SEP amplitudes, and spinal SEP amplitudes were predicted by peripheral NAP amplitudes ($\beta_{ESG} = 0.03$, $t(145171.5) = 10.01$, $p < 0.001$ and $\beta_{periphery} = 0.02$, $t(142032.7) = 8.40$, $p < 0.001$). Adding the factor *stimulation condition* to the models revealed that both these relationships were driven by effects of the type of stimulation on cortical SEP amplitudes ($\beta_{finger1} = 0.72$, $t_{finger1}(145163.8) = 98.35$, $\beta_{finger2} = 0.62$, $t_{finger2}(145185.0) = 89.15$, $\beta_{fingers1\&2} = 0.47$, $t_{fingers1\&2}(145185.0) = 66.99$, all $p < 0.001$), as well as on spinal SEP amplitudes ($\beta_{finger1} = 0.21$, $t_{finger1}(136741.6) = 26.11$, $\beta_{finger2} = 0.19$, $t_{finger2}(141498.6) = 24.22$, $\beta_{fingers1\&2} = 0.15$, $t_{fingers1\&2}(141288.6) = 19.87$, all $p < 0.001$); effect contrasts with reference level *mixed nerve stimulation*. At the same time, the effects of spinal SEP on cortical SEP amplitude and of peripheral NAP amplitude on spinal SEP amplitude were no longer significant and thus fully explained by *stimulation condition* ($\beta_{ESG} = 0.001$, $t(145177.4) = 0.72$, $p = 0.47$ and $\beta_{periphery} = -0.00$, $t(142364.2) = -0.42$, $p = 0.672$). Hence, *finger1*, *finger2*, as well as *fingers1&2* stimulations all resulted in differential amplitudes as compared to *mixed nerve* stimulation, both on the spinal as well as on the cortical level, and this amplitude variance was fully shared among the processing levels, explaining single-trial covariation across periphery, spinal cord and cortex.

A similar picture emerged for foot stimuli: cortical SEP amplitudes were predicted by spinal SEP amplitudes ($\beta_{ESG} = -0.04$, $t(151307.0) = -14.38$, $p < 0.001$) and spinal SEP amplitudes were predicted by peripheral NAP amplitudes ($\beta_{periphery} = 0.02$, $t(151223.8) = 7.94$, $p < 0.001$) when not controlling for stimulation conditions; please note that the negative sign of β_{ESG} reflects the fact that spinal SEP amplitudes are measured as negative potentials while the first cortical SEP in the foot region, the P40, is a positive peak. When adding the factor *stimulation condition*, again, all types of stimulation affected the cortical level ($\beta_{toe1} = -0.61$, $t_{toe\ 1}(151264.6) = -86.60$, $\beta_{toe2} = -0.50$, $t_{toe2}(151264.8) = -70.15$, $\beta_{toes1\&2} = -0.32$, $t_{toes1\&2}(151268.8) = -46.32$, all $p < 0.001$) as well as the spinal level ($\beta_{toe1} = 0.33$, $t_{toe\ 1}(140802.2) = 43.82$, $\beta_{toe2} = 0.33$, $t_{toe2}(140806.3) = 43.51$, $\beta_{toes1\&2}$ =

.26, $t_{toes1\&2}$(141379.1) = 35.86, all $p < 0.001$). While the factor *stimulation condition* fully accounted for the effect of peripheral NAP on spinal amplitude, which was no longer existent ($\beta_{periphery}$ = 0.00, t(151323.8) = 0.00, $p > 0.99$), a main – though slightly attenuated – effect of spinal amplitude on cortical amplitude still remained (β_{ESG} = -0.02, t(151320.4) = -3.72, $p < 0.001$). Additionally, small interaction effects on cortical amplitudes emerged between spinal amplitudes and toe2 stimulation, $\beta_{ESG * toe 2}$ = 0.02, $t_{ESG * toe2}$(151314.1) = 3.02, $p_{ESG * toe2}$ = 0.003, as well as between spinal amplitudes and toes 1 & 2 stimulation, $\beta_{ESG * toes1\&2}$ = 0.02, $t_{ESG * toes1\&2}$(151314.5) = 2.99, $p_{ESG * toes1\&2}$ = 0.003.

Taken together, the effects of different stimulation types (i.e., *mixed nerve, finger1/toe1*, *finger2/toe2*, *fingers1&2/toes1&2*) seem to propagate through the somatosensory processing hierarchy, jointly affecting the amplitudes of peripheral NAPs, spinal cord responses, and initial cortical potentials in the primary somatosensory cortex. This observation applied to both hand and foot stimulation, though with additional effects of spinal amplitudes on cortical amplitudes beyond the effect of stimulation condition in foot stimuli.

Placement of recording reference

While other ESG studies have also used our choice of reference position for the recording of cervical and lumbar SEPs [1–9], the choice of reference position is always a compromise. For spinal recordings a non-cephalic reference as used here is generally suggested, but studies often use different references for cervical and lumbar recordings, such as the acromion for cervical or the pelvic bone for lumbar recordings. We wanted to use a reference position that is i) not lateralized, ii) ideal for both cervical and lumbar recordings and iii) positioned on a bone (not on muscle) and thus selected the spinous process of the 6th thoracic vertebra after running several pilot recordings with different reference positions.

References

1. Beric A. Stability of lumbosacral somatosensory evoked potentials in a long-term follow-up. Muscle Nerve. 1988;11: 621–626. doi:10.1002/mus.880110615
2. Delbeke J, McComas AJ, Kopec SJ. Analysis of evoked lumbosacral potentials in man. J Neurol Neurosurg Psychiatry. 1978;41: 293–302. doi:10.1136/jnnp.41.4.293
3. Dimitrijevic MR, Larsson LE, Lehmkuhl D, Sherwood A. Evoked spinal cord and nerve root potentials in humans using a non-invasive recording technique. Electroencephalogr Clin Neurophysiol. 1978;45: 331–340. doi:10.1016/0013-4694(78)90185-2
4. Dimitrijevic MR, Lehmkuhl LD, Sedgwick EM, Sherwood AM, McKay WB. Characteristics of spinal cord-evoked responses in man. Appl Neurophysiol. 1980;43: 118–127. doi:10.1159/000102245
5. Gilmore RL, Bass NH, Wright EA, Greathouse D, Stanback K, Norvell E. Developmental assessment of spinal cord and cortical evoked potentials after tibial nerve stimulation: effects of age and stature on normative data during childhood. Electroencephalogr Clin Neurophysiol. 1985;62: 241–251. doi:10.1016/0168-5597(85)90002-4
6. Lastimosa AC, Bass NH, Stanback K, Norvell EE. Lumbar spinal cord and early cortical evoked potentials after tibial nerve stimulation: effects of stature on normative data. Electroencephalogr Clin Neurophysiol. 1982;54: 499–507. doi:10.1016/0013-4694(82)90035-9
7. Maccabee PJ, Pinkhasov EI, Cracco RQ. Short latency somatosensory evoked potentials to median nerve stimulation: effect of low frequency filter. Electroencephalogr Clin Neurophysiol. 1983;55: 34–44. doi:10.1016/0013-4694(83)90144-x
8. McKay WB, Galloway BL. Technological Aspects of Recording Evoked Potentials from the Cauda Equina and Lumbosacral Spinal Cord in Man. Am J EEG Technol. 1979;19: 83–96. doi:10.1080/00029238.1979.11079968
9. Ratto S, Abbruzzese M, Abbruzzese G, Favale E. Surface recording of the spinal ventral root discharge in man. An experimental study. Brain. 1983;106 (Pt 4): 897–909. doi:10.1093/brain/106.4.897
