# Supplementary figures and images for: A multichannel electrophysiological approach to noninvasively and precisely record human spinal cord activity
Source: PLoS Biol. 2024 Oct 31;22(10):e3002828. doi: 10.1371/journal.pbio.3002828 (PMC11527246; doi:10.1371/journal.pbio.3002828)

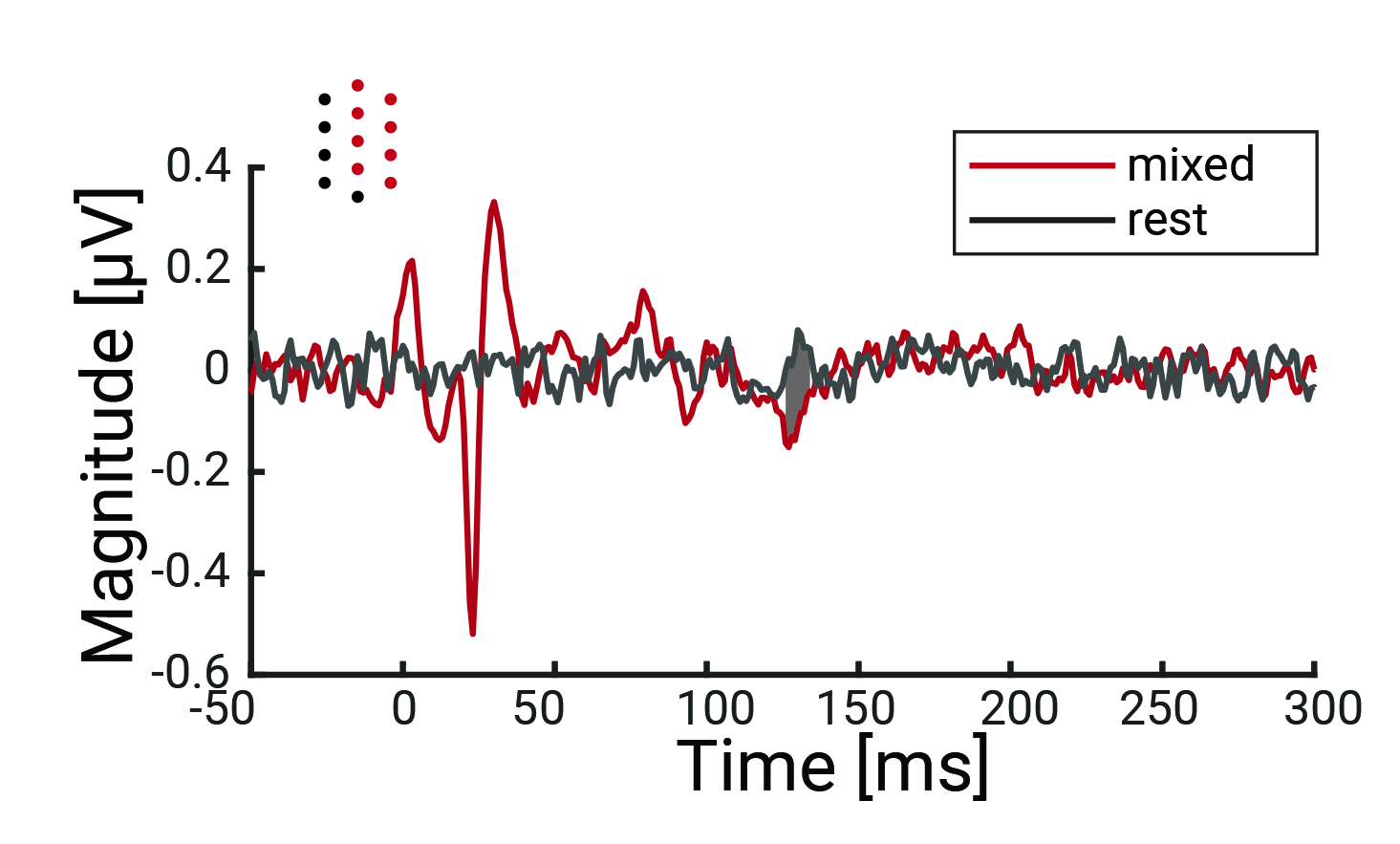

Supplement: S1 Fig — Grand-average over all participants in the foot-mixed condition and in simulated epochs from rest data. The plotted signal is an average over all channels that are part of the identified cluster (channels displayed as red dots on the top left). The gray area between 126–132 ms identifies the time range in which the 2 signals are statistically different; note that this result did not replicate in Experiment 2. (TIF) [file pbio.3002828.s002.tif]
