# Supplementary material for: A multichannel electrophysiological approach to noninvasively and precisely record human spinal cord activity
Source: PLoS Biol. 2024 Oct 31;22(10):e3002828. doi: 10.1371/journal.pbio.3002828 (PMC11527246; doi:10.1371/journal.pbio.3002828)
Supplement: S1 Table — Group-level descriptive statistics for SEP- and NAP-amplitudes, latencies and SNR (mean and standard error of the mean) and one-sample t test of SEP- and NAP-amplitudes in the hand-mixed and foot-mixed conditions of Experiment 2 (N = 24). Note that we only focused on the major peripheral, spinal, and cortical components here for replication purposes and thus do not report Erb’s point and brainstem potentials. Abbreviations: vr = ventral reference, tr = thoracic reference, CCA–canonical correlation analysis, SEP = somatosensory evoked potential, NAP = nerve action potential, # = number of participants in which potential was visible at the individual level, SNR = signal-to-noise ratio). (PDF) [file pbio.3002828.s003.pdf]

SEP / NAP	#	Latency [ms]	Amplitude [µV / a.u.]	SNR	tstat	P	95%-CI	Cohen's d
Mixed median nerve stimulation (hand-mixed)								
N6	24	6.46 ± 0.10	-2.61 ± 0.29	36.52 ± 12.23	-8.93	<0.001	[-3.21; -2.01]	-1.82
N13 (tr)	24	13.46 ± 0.20	-0.86 ± 0.07	9.37 ± 1.51	-12.31	<0.001	[-1.01; -0.72]	-2.51
N13 (vr)	24	13.75 ± 0.17	-1.38 ± 0.09	14.36 ± 1.98	-16.09	<0.001	[-1.55; -1.20]	-3.28
N13 (CCA)	24	13.58 ± 0.19	-0.39 ± 0.04	24.01 ± 3.64	-10.40	<0.001	[-0.46; -0.31]	-2.12
N20 (CCA)	24	19.79 ± 0.17	-1.10 ± 0.08	24.07 ± 2.28	-13.80	<0.001	[-1.26; -0.93]	-2.82
Mixed tibial nerve stimulation (foot-mixed)								
N8	22	9.54 ± 0.16	-0.99 ± 0.16	13.30 ± 4.49	-6.20	<0.001	[-1.32; -0.66]	-1.27
N22 (tr)	24	24.21 ± 0.36	-0.57 ± 0.07	6.20 ± 1.07	-8.38	<0.001	[-0.71; -0.43]	-1.71
N22 (vr)	24	24.71 ± 0.43	-0.48 ± 0.06	10.09 ± 1.70	-8.53	<0.001	[-0.59; -0.36]	-1.74
N22 (CCA)	24	24.25 ± 0.32	-0.48 ± 0.05	24.97 ± 5.66	-9.46	<0.001	[-0.58; -0.37]	-1.93
P40 (CCA)	24	40.92 ± 0.58	1.17 ± 0.09	27.93 ± 3.07	12.80	<0.001	[0.98; 1.36]	2.62
