# Supplementary material for: A multichannel electrophysiological approach to noninvasively and precisely record human spinal cord activity
Source: PLoS Biol. 2024 Oct 31;22(10):e3002828. doi: 10.1371/journal.pbio.3002828 (PMC11527246; doi:10.1371/journal.pbio.3002828)
Supplement: S2 Table — Group-level descriptive statistics for SEP- and NAP-amplitudes, latencies and SNR (mean and standard error) and one-sample t test of SEP- and NAP-amplitudes in all hand-sensory and foot-sensory conditions of Experiment 2 (vr = ventral reference, tr = thoracic reference, CCA = canonical correlation analysis, # = number of participants with potentials visible at the individual level). (PDF) [file pbio.3002828.s004.pdf]

SEP / NAP	#	Latency [ms]	Amplitude [µV / a.u.]	SNR	tstat	p	95%-CI	Cohen's d
		Sensory median nerve stimulation (hand-sensory)						
		Index finger (finger1)						
N6	23	10.29 ± 0.15	-0.34 ± 0.04	9.90 ± 1.82	-8.32	<0.001	[-0.43; -0.26]	-1.7
N13 (tr)	21	18.13 ± 0.26	-0.19 ± 0.04	2.36 ± 0.35	-5.02	<0.001	[-0.26; -0.11]	-1.03
N13 (vr)	23	18.13 ± 0.24	-0.25 ± 0.04	3.33 ± 0.59	-6.38	<0.001	[-0.33; -0.17]	-1.30
N13 (CCA)	21	18.17 ± 0.20	-0.09 ± 0.01	3.50 ± 0.68	-5.71	<0.001	[-0.12; -0.05]	-1.16
N20 (CCA)	24	23.79 ± 0.23	-0.34 ± 0.04	17.08 ± 3.73	-9.16	<0.001	[-0.41; -0.26]	-1.87
		Middle finger (finger2)						
N6	23	10.17 ± 0.14	-0.41 ± 0.05	9.42 ± 1.56	-8.91	<0.001	[-0.50; -0.31]	-1.82
N13 (tr)	23	17.92 ± 0.28	-0.24 ± 0.06	2.28 ± 0.45	-3.84	0.001	[-0.38; -0.11]	-0.78
N13 (vr)	23	17.75 ± 0.25	-0.42 ± 0.06	3.31 ± 0.43	-7.35	<0.001	[-0.53; -0.30]	-1.50
N13 (CCA)	24	17.79 ± 0.29	-0.12 ± 0.01	4.90 ± 1.24	-8.60	<0.001	[-0.15; -0.09]	-1.76
N20 (CCA)	24	23.71 ± 0.26	-0.41 ± 0.04	22.29 ± 6.40	-9.59	<0.001	[-0.50; -0.32]	-1.96
		Index and middle finger (fingers1&2)						
N6	23	10.13 ± 0.13	-0.76 ± 0.08	11.34 ± 1.84	-9.51	<0.001	[-0.93; -0.60]	-1.94
N13 (tr)	22	17.38 ± 0.27	-0.39 ± 0.05	4.36 ± 1.52	-8.56	<0.001	[-0.49; -0.30]	-1.75
N13 (vr)	22	17.58 ± 0.22	-0.61 ± 0.07	6.37 ± 1.11	-8.89	<0.001	[-0.75; -0.47]	-1.81
N13 (CCA)	24	17.58 ± 0.25	-0.16 ± 0.02	6.76 ± 1.89	-9.09	<0.001	[-0.20; -0.13]	-1.86
N20 (CCA)	24	23.71 ± 0.24	-0.58 ± 0.06	42.14 ± 15.78	-9.92	<0.001	[-0.70; -0.46]	-2.02
		Sensory tibial nerve stimulation (foot-sensory)						
		First toe (toe1)						
N8	20	15.46 ± 0.28	-0.11 ± 0.02	4.05 ± 0.82	-6.33	<0.001	[-0.14; -0.07]	-1.29
N22 (tr)	24	31.21 ± 0.60	-0.17 ± 0.03	2.39 ± 0.69	-6.63	<0.001	[-0.23; -0.12]	-1.35
N22 (vr)	24	31.25 ± 0.60	-0.10 ± 0.02	1.72 ± 0.26	-4.51	<0.001	[-0.14; -0.05]	-0.92
N22 (CCA)	22	31.38 ± 0.52	-0.10 ± 0.01	3.61 ± 0.60	-7.03	<0.001	[-0.13; -0.07]	-1.44
P40 (CCA)	24	49.83 ± 0.71	0.53 ± 0.07	26.84 ± 10.81	7.13	<0.001	[0.38; 0.68]	1.46
		Second toe (toe2)						
N8	20	15.71 ± 0.29	-0.10 ± 0.02	5.49 ± 1.99	-6.32	<0.001	[-0.13; -0.07]	-1.29
N22 (tr)	23	31.25 ± 0.58	-0.21 ± 0.04	2.15 ± 0.32	-5.78	<0.001	[-0.28; -0.13]	-1.18
N22 (vr)	23	31.04 ± 0.62	-0.08 ± 0.02	2.81 ± 0.73	-3.13	0.004	[-0.13; -0.03]	-0.64
N22 (CCA)	23	31.38 ± 0.49	-0.10 ± 0.01	4.20 ± 0.61	-8.64	<0.001	[-0.13; -0.08]	-1.76
P40 (CCA)	23	50.42 ± 0.75	0.62 ± 0.08	26.84 ± 5.50	7.43	<0.001	[0.44; 0.79]	1.52
		First and second toe (toes1&2)						
N8	19	15.33 ± 0.28	-0.19 ± 0.03	9.29 ± 2.92	-6.95	<0.001	[-0.25; -0.13]	-1.42
N22 (tr)	23	31.21 ± 0.60	-0.22 ± 0.02	3.87 ± 0.76	-9.38	<0.001	[-0.26; -0.17]	-1.91
N22 (vr)	23	31.00 ± 0.56	-0.18 ± 0.03	3.40 ± 0.72	-5.27	<0.001	[-0.25; -0.11]	-1.08
N22 (CCA)	23	31.38 ± 0.48	-0.18 ± 0.02	7.59 ± 1.72	-8.44	<0.001	[-0.22; -0.14]	-1.72
P40 (CCA)	23	49.25 ± 0.73	0.81 ± 0.10	26.72 ± 5.66	8.42	<0.001	[0.61; 1.01]	1.72
