# Supplementary material for: A multichannel electrophysiological approach to noninvasively and precisely record human spinal cord activity
Source: PLoS Biol. 2024 Oct 31;22(10):e3002828. doi: 10.1371/journal.pbio.3002828 (PMC11527246; doi:10.1371/journal.pbio.3002828)
Supplement: S3 Table — Paired t test for the comparisons between hand-mixed and fingers1&2 conditions or foot-mixed and toes1&2 conditions. Tested were the amplitudes and the latencies of the SEPs and peripheral NAPs. Data come only from Experiment 2 (vr = ventral reference, tr = thoracic reference, CCA = canonical correlation analysis). (PDF) [file pbio.3002828.s005.pdf]

SEP / NAP	tstat	p	95%-CI	Cohen's d
Amplitude: Hand-mixed – fingers1&2				
N6	-6.73	<0.001	[-2.41; -1.28]	-1.37
N13 (tr)	-5.38	<0.001	[-0.65; -0.29]	-1.10
N13 (vr)	-7.42	<0.001	[-1.14; -0.64]	-1.52
N13 (CCA)	-9.56	<0.001	[-0.27; -0.17]	-1.95
N20 (CCA)	-10.32	<0.001	[-0.62; -0.41]	-2.11
Latency: Hand-mixed – fingers1&2				
N6	-28.20	<0.001	[-3.94; -3.40]	-5.76
N13 (tr)	-18.10	<0.001	[-4.36; -3.47]	-3.70
N13 (vr)	-18.10	<0.001	[-4.36; -3.47]	-3.70
N13 (CCA)	-21.01	<0.001	[-4.39; -3.61]	-4.29
N20 (CCA)	-32.88	<0.001	[-4.16; -3.67]	-6.71
Amplitude: Foot-mixed – toes1&2				
N8	-5.35	0.001	[-1.09; -0.48]	-1.09
N22 (tr)	-5.50	<0.001	[-0.49; -0.22]	-1.12
N22 (vr)	-5.08	<0.001	[-0.46; -0.19]	-1.04
N22 (CCA)	-7.18	<0.001	[-0.38; -0.21]	-1.47
P40 (CCA)	4.00	0.001	[0.17; 0.55]	0.82
Latency: Foot-mixed – toes1&2				
N8	-24.46	<0.001	[-6.24; -5.26]	-4.99
N22 (tr)	-18.86	<0.001	[-7.86; -6.31]	-3.85
N22 (vr)	-18.86	<0.001	[-7.86; -6.31]	-3.85
N22 (CCA)	-20.82	<0.001	[-7.83; -6.42]	-4.25
P40 (CCA)	-18.56	<0.001	[-9.26; -7.40]	-3.79
