# Supplementary material for: A multichannel electrophysiological approach to noninvasively and precisely record human spinal cord activity
Source: PLoS Biol. 2024 Oct 31;22(10):e3002828. doi: 10.1371/journal.pbio.3002828 (PMC11527246; doi:10.1371/journal.pbio.3002828)
Supplement: Attachment — Submitted filename: ResponseToReviewers.pdf [file pbio.3002828.s013.pdf]


Reviewer #1: This is an important paper which outlines electrophysiological recordings along the complete neuro-axis from peripheral nerve, through brainstem and to primary sensor cortex. It highlights the quality of work that can be done with standard and affordable electrophysiological measures. This represents a huge amount of recording time and number of subjects in a well-designed paradigm. I do have one major concern, that is hopefully a misunderstanding, and that concerns the CCA analysis.

We would like to thank this reviewer for their positive evaluation and their helpful comments, which have considerably improved the manuscript.

Firstly, as the authors point out, there is the user selection of the time-period and then a choice of the significant CCA component. This adds to some loss of objectivity. This should be noted in the limitations section.

We agree with this point but would also like to mention that the selection of CCA components was based on a priori knowledge about the latency of evoked responses and the expectation that the most relevant components should have the highest CCA correlation. Pre-selection of time and frequency parameters for multivariate approaches is quite common in EEG literature (Blankertz et al., 2007; Michel & Murray, 2012) and can be further verified with validation analyses (such as those now carried out in the present study in response to this reviewer's comments; see below). A totally automated procedure could be developed in the future, yet some predefined criteria should still be considered. We have therefore added the following sentence to the limitations section of the Discussion (L660-663): "Finally, it should also be noted that there is some loss of objectivity when using CCA, considering that a-priori knowledge informed the time-period for training CCA and the choice of component – this might be alleviated in the future by developing automated procedures based on pre-defined criteria.".

We would also like to note that the selected component was always present within the first two components and have added the relevant information to the Methods section. Study 1 (L1009-1014): "As expected, the selected component was present in all participants among the first two CCA components, i.e., those with the largest canonical correlation coefficients: for spinal data, we selected the first component in every participant (median first component: N = 36; tibial first component: N = 36) and for cortical data, we nearly always selected the first component (median first component: N = 32; median second component: N = 4; tibial first component: N = 35; tibial second component: N = 1).". Study 2 (L1133-1138): "The selected component was present in all participants among the first two CCA components, i.e., those with the largest canonical correlation coefficients: for spinal data, we selected the first component in every participant (median first component: N = 24; tibial first component: N = 24) and for cortical data, we nearly always selected the first component (median first component: N = 20; median second component: N = 4; tibial first component: N = 22; tibial second component: N = 2)."

As the time-window was user specified, please make it clear how SNR was calculated (i.e. if you choose the peak to optimize, and then measure SNR at that peak is there some circularity).

CCA training was based on an 11ms long time-window centred on the latency of the response of interest obtained individually via the peak latency of the target channel over

the 6th cervical or 1st lumbar vertebra, respectively. SNR was then calculated in exactly the same way for single-channel data and CCA data, namely by individual peak-identification and then defining signal (root-mean-square extracted in a time window of +/-1 ms around the individual peak latency) and noise (root-mean-square extracted in the same time window before the stimulus onset). Considering that single-channel data informed CCA training and that SNR calculation was identical between both types of data, there is no circularity that would artificially inflate CCA-SNR. The window-length for CCA training is mentioned in L995-998: "Since we were interested in early components of the SEP, we only subjected a short time window to CCA (and not the whole epoch length), namely a window from 5 ms before to 5ms after the peak of the cortical or spinal SEP component of interest (resulting in 11 data points per trial).". The SNR window-length is mentioned in L1065-1067: "For all potentials, the SNR was quantified as the root-mean-square of the signal (extracted in a in a time window of +/-1 ms around the individual peak latency) divided by the root-mean-square of the noise (extracted in the same time window before the stimulus onset).".

Please make it clear how CCA was used in the Interaction ratio section. For example, it would seem that amplitudes can only be compared if the same linear (CCA) mixture is used for all comparisons (rather than a different CCA mixture for each component).

We would like to thank the reviewer for bringing up this important point, as indeed the choice of component for the Interaction Ratio analysis was performed in a way that avoids the problem that this reviewer mentions: i) CCA was trained on mixed-nerve trials from Experiment 2, ii) the relevant component was then chosen based on this mixed nerve training, and iii) this spatial filter was then applied to all the sensory nerve trials (finger1, finger2, finger12), thus ensuring that there is no selection bias as the comparison was carried out using the same spatial filter for all conditions. To clarify this, we have now added the following sentence to the Results section (L349-352): "CCA training and component selection was based on mixed nerve data (which have a higher SNR than sensory nerve data) and the chosen spatial filter was then applied to all sensory nerve conditions, ensuring independence of selection and testing."

Secondly, could the authors please be more explicit with the notation on page 22. Please give the dimensions of X and Y and the wx, wy in terms of rows and columns. Also, please specify Y in terms of X. As far as I understand Y is a repetition of a specific latency range of mean(X) over Ntrials.

We have updated the paragraph on CCA, providing the requested details in the hope that it is easier to follow now (L979-999): "We employed a variant of CCA as used previously for single-trial extraction in EEG data (Fedele et al., 2013; Waterstraat et al., 2015; Stephani et al., 2021), also known as canonical correlation average regression (Waterstraat et al., 2015). For two multi-channel signals X and Y, CCA finds the spatial filters w_x and w_y that maximize the correlation

$$\max_{w_x, w_y} corr\left(w_x^T X, w_y^T Y\right).$$

While both multi-channel matrices X and Y have the same size with the structure channel × time, X is a multi-channel signal that contains all concatenated epochs from 1 to N and Y is a signal that contains N times the average over all epochs concatenated (with N being the number of all epochs from one participant's recording); in other words, Y is

the same size as X, only that instead of single trials (as in the case of X) it is made up of repetitions of the average of all trials, again using the same latency range as in X. More precisely, both X and Y are of size [number of channels × number of samples] and both w_x and w_y are of size [number of channels × number of channels] (in the case of full rank), with "number of channels" being 64 for EEG and 17 for ESG and "number of samples" being N (2000 in case of no trial rejection) * 11 (see below for rationale). Applied in this way, the CCA procedure serves as a template matching between the single-trial and the average of all trials. The spatial filter w_x corresponds to a spatial weighting of the multi-channel signal to separate SEP-related activity from background noise (Stephani et al., 2021). Since we were interested in early components of the SEP, we only subjected a short time window to CCA (and not the whole epoch length), namely a window from 5 ms before to 5ms after the peak of the cortical or spinal SEP component of interest (resulting in 11 data points per trial). The extracted spatial filter was then applied to the whole length of the epochs."

Then also please specify (explicitly in equation) how the canonical component of interest is created. My confusion is that normally one would have more (at least 4 times) trials (rows) than features (columns). My understanding is that, by definition, one would expect a very high and significant canonical correlations otherwise. Again, hopefully adding more information (like effective degrees of freedom and as many other details of the CCA as possible etc) should remove these concerns.

Following this reviewer's suggestion, we have added more details on CCA (specifically regarding the motivation of using this technique in the context of electrophysiological data analysis and the trial-to-feature ratio) to hopefully remove their concerns. First addition (L974-979): "In the context of EEG, CCA has for example been used as blind source separation approach to remove noise such as muscle activity (deClercq et al., 2006) and as a technique to improve single-trial classification of evoked potentials (Spüler et al., 2014). In both cases, the goal is to obtain a spatial filter and consequently a projected component with the largest similarity between two data matrices. Inverting a spatial filter creates corresponding topographies which can then be interpreted in a neurophysiologically meaningful manner (Haufe et al., 2014)." Second addition (L1021-1028): "Last but not least, it is important to note that for such a multivariate analysis the number of samples should in principle be at least ten times the number of variables (Mandeville & Roscoe, 1971), though more recent efforts also taking into account the effect size suggest an even larger sample-to-feature ratio: e.g. in the case of a between-set correlation of 0.3 (close to the average canonical correlations we observed: 0.25 for median and 0.29 for tibial nerve stimulation) at least 50 samples per feature (Helmer et al., 2024). In our case, we far exceed the suggested sample-to-feature ratio due to very large number of trials used for training (i.e. in the case of no trial rejections, 2000 trials with 11 data points each compared to 64 (EEG) or 17 (ESG) channels).". In addition, we provide new analyses below showing that our results are unlikely to be produced by overfitting related to CCA.

One way to show this is less of a worry empirically could be to replace the raw data X with the resting state data (as used elegantly to demonstrate the late component in figure 3G). Ideally this would not show a significant component.

We have followed this excellent suggestion and have added the following section to the Results (L276-293): "Furthermore, in order to demonstrate that CCA is not creating artefactual signal due to overfitting, we carried out a control analysis. More specifically,

in each participant we i) trained CCA on a random selection of 50% of the trials (underlying data: band-pass filtered, anterior-electrode re-referenced, epoched; time windows: 8-18ms for median and 14.5-29.5 for tibial nerve stimulation;), ii) saved the time-course of the first component, iii) repeated this procedure a thousand times and iv) then calculated all pair-wise absolute correlations between the obtained component time-courses (in the CCA training time-window). This procedure was also carried out on resting-state data, using identical trial timings. At the group level, we then compared the correlation strength between task-based data and resting-state data via a paired t-test. The main idea of this procedure was to demonstrate that correlations between subsampled CCA components would be substantially stronger in the presence of repeated evoked responses compared to CCA performed on the data from the resting-state data where we do not expect repeated evoked responses. For median nerve stimulation, we obtained a group-average absolute correlation of 0.98 (range across participants: 0.76 – 1) in the task-based data and a group-average absolute correlation of 0.58 (range across participants: 0.46 – 0.73) in the resting-state data; for tibial nerve stimulation, the respective values were 0.96 (range: 0.61 – 1) for task and 0.50 (range: 0.37 – 0.68) for rest. Importantly, component correlations were significantly higher in task-based data than in resting-state data (median nerve stimulation: $t = 27.80$, $p < 9.5e\text{-}26$; tibial nerve stimulation: $t = 25.06$, $p < 3.1e\text{-}24$; one-tailed).".

Minor.

Figure 1. Please put number of subjects that went into grand average into figure caption.

We assume that this reviewer is referring to Figure 2 (considering that Figure 1 does not contain data) and have thus added the number of participants (N = 36) both to the text describing this figure as well as to the figure legend.

Reviewer #2: The paper shows the optimization of a methodology to record spinal cord activities in a non-invasive way. The demonstration of the efficacy of the approach is well-designed and the results quite convincing.

We would like to thank this reviewer for their positive evaluation. We have followed their recommendations by adding data from a third experiment to the manuscript in order to highlight the usability of our approach.

However, I found a bit limited the overall investigation of the real usability of this approach. I would suggest the authors to add some experiments with more "ecological" touch or proprioceptive stimuli of hand and foot... what is the overall ability to discriminate these sensations?

We appreciate this reviewer's suggestions to highlight the usability of our approach. We would like to mention however that this article was not submitted as a 'Research article' to PLOS Biology, but as a 'Methods and resources article', and is thus largely focussed on demonstrating the improvements of our approach over previously established methods (as per journal guidelines). In addition, Experiment 2 was already designed to serve as a demonstration of this approach's power to reveal neurobiological phenomena of interest, such as clearly detectable spinal cord potentials after single-finger stimulation, where only a very limited number of myelinated nerve fibres is stimulated (estimated to be roughly between 2000 and 4000 for a single finger; Corniani & Saal, 2020) and the bottom-up signal integration delineated in the manuscript.

Nevertheless, in order to further demonstrate the applicability of our approach, we have followed this reviewer's suggestion and have added new data from a third experiment to the manuscript: here, we have not focussed on touch or proprioception, since both mechanosensory (e.g. Pratt et al., 1979; Pratt & Star, 1981) and proprioceptive spinal processes (e.g. Tarkka & Teräväinen, 1985; Reni et al., 1988) have already been investigated. Instead, we have added a non-invasive proof-of-principle study (N = 7) demonstrating the first in-human electrophysiological recording of nociceptive responses in the spinal cord underlying heat-pain. There are several distinct aspects to this approach that are worth highlighting. First, these responses are generated via a more 'ecological' pathway (thermal nociceptive stimulation acts on specific receptors instead of bypassing them as our electrical stimulation does), in line with this reviewer's suggestion. Second, in contrast to touch or proprioception (or electrical stimulation for that matter), heat pain responses as observed here are mediated via a different type of afferent fibres (A-delta) that present with lower and more variable conduction velocity and thus a much lower synchronicity and resulting SNR at the spinal level – demonstrating that our multi-channel approach can reveal such responses highlights its 'real usability'. Finally, in contrast to Experiment 1 and Experiment 2 in our manuscript (which were carried out with participants lying down and electrophysiological data being recorded in a shielded chamber), the newly added data were obtained in less favourable (but more realistic) recording settings: sitting upright (adding myogenic noise) and without any shielding (adding environmental noise).

We have added all the relevant information regarding this third experiment to the manuscript and have also copied this here:

Abstract (L060-061): “Finally, we demonstrate the versatility of this approach by providing the first in-human non-invasive recordings of nociceptive spinal cord responses during heat-pain stimulation.”

Introduction (L152-154): “In a final proof-of-principle experiment, we furthermore assess the possibility of using this non-invasive approach to detect nociceptive spinal cord responses in humans.”

Results (L432-442): “In a final proof-of-principle experiment (N=7), we aimed to provide an example of the usability of this approach by recording spinal cord – and simultaneously also cortical – responses to nociceptive heat-pain stimulation (induced via a CO_2-laser). At the group level, we observed the canonical laser-evoked potentials (LEPs), i.e. the cortical N1 and N2P2 components, with the expected latency and topography (Figure 8a). Most importantly, we also observed a distinct LEP at the spinal level, consisting of a negative deflection at 52ms (Figure 8b). This response could only be obtained by making use of our multi-channel set-up and spatial filtering approach, since it could not be detected in single electrode signals. Notably, this group-level response was consistent across data splits (four-fold split depicted in Figure 8c) and observed – with slight latency jitter – in every single participant (Figure 8d).”

Discussion (L582-598): “In a final experiment, we demonstrated the versatility of our non-invasive approach by providing corticospinal electrophysiological recordings of responses to nociceptive stimulation in a heat-pain paradigm. While there is a multitude of EEG studies assessing cortical responses to various forms of nociceptive stimuli (e.g. laser evoked potentials [LEPs; Plaghki & Mouraux, 2005], contact heat evoked potentials [CHEPS; Lejeune et al., 2023], pinprick evoked potentials [PEPs; Iannetti et al. 2013]), a non-invasive assessment of spinal responses to any type of nociceptive stimulation has hitherto not been reported to our knowledge. In a first proof-of-principle experiment, we therefore leveraged the sensitivity increase afforded by our multi-channel set-up to demonstrate concurrently recorded cortical and spinal LEPs. The spinal response occurred at a time-point consistent with the activation of nociceptive A-delta fibres and was observable to varying degrees in every single participant after spatial filtering.

While this first demonstration of non-invasively recorded spinal LEPs obviously awaits replication, it is a promising step to investigate entire CNS mechanisms underlying the experience of pain in health and disease. A non-invasive and direct window into spinal nociceptive processing is highly relevant for pain research, considering that the spinal cord is not only the first CNS processing station for nociceptive stimuli (Basbaum et al., 2009), but also a target of powerful descending control mechanisms (Heinricher et al., 2009) and a structure often implicated in pain chronification (Kuner, 2015).”

Methods (L1197-1292): “*Participants.* We acquired data from seven healthy volunteers (5 female; mean age: 30.6 years, range: 23-36 years), all of whom provided written informed consent. The study was approved by the Ethics Committee at the Medical Faculty of the University of Leipzig

Laser stimulation. Individually calibrated painful heat stimuli (duration 125ms) were delivered to the dorsum of left hand using a CO_2-laser with a with a wavelength of 10.6μm and a beam diameter of 6mm (LSD; Laser Stimulation Device, SIFEC s.a., Ferrières, Belgium). The LSD contains a closed loop temperature control system to maintain constant skin temperature during stimulation by adjusting the energy output. The stimulus position was controlled by an electric motor moving the laser head relative to a participant’s hand, allowing for precise control of stimulation position. Throughout the entire experiment, participants wore protective goggles.

Experimental design. The here-reported data are part of a larger experiment also involving other stimulation modalities, but we solely focus on laser stimulation in this report. Before any electrodes were attached to the participant, the experiment started with a calibration procedure in order to find temperatures that would be perceived as clearly painful, but tolerable (mean temperature: 55.9°C; range: 53-59°C). There were 10 blocks of laser stimulation (with a break of ~5-10 minutes between blocks), with each block containing 36 stimuli, separated by an ISI of 1.53s with a jitter between +/-100ms (drawn from a uniform distribution). In each block, the laser beam was shifted over the dorsum of the left hand in an S-shaped pattern along a 6x6 grid (size 5x5cm): the start could be in any of the 4 corners of the grid and the laser would always move along the rows in the anterior/posterior direction before moving to the next column, until all 36 cells had been stimulated once.

Electrographic recordings. ECG, EEG, EOG, and ESG data were acquired using the same equipment as described in Experiment 1 and Experiment 2. ECG data were recorded via an electrode placed on the left costal arch, referenced to an electrode placed underneath the right clavicular. EEG data were recorded via a standard 32-channel montage according to the 10-20 system and referenced to the nose. EOG data were recorded via two additional electrodes placed on the canthus of the right eye (referenced to nose) and below the right eye (referenced to Fp2). ESG recordings were again based on a custom-made electrode patch (consisting of the same fabric as the EEG cap), but now with a higher electrode number than in Experiment 1 and Experiment 2 and focused solely on the cervical spinal cord. The patch consisted of 38 electrodes centred around an electrode over the spinous process of the 7th cervical vertebra. The midline of this electrode-grid was positioned vertically along the spine and consisted of 7 electrodes (the 4th one being centered on vertebra C7) with a vertical inter-electrode distance of 2 cm. Two further vertical lines of 6 electrodes each were placed 1.5 cm to the right and left of the midline electrodes, another two vertical lines of 5 electrodes were placed 3 cm to the right and left of the midline, and another two vertical lines of 2 electrodes each were placed 5 cm to the right and left of the midline. Additional electrodes were placed on the 1st cervical vertebra and on the inion. In addition to the dorsal electrodes, there were also 3 ventral electrodes at the anterior neck (one supra-glottic electrode (CA1), one above the suprasternal notch (CA3) and the third one in the middle between these two (CA2)). ESG data were referenced to an electrode positioned over the spinous process of the 6th thoracic vertebra (Th6). The active ground electrode stabilized the signal via the "driven right leg" principle. It was placed at POz in the EEG montage and on the spinous process of the 10th thoracic vertebra in the ESG montage.

Data analysis – EEG. All analyses were performed using Python 3.10 and MNE (https://mne.tools/stable/index.html; version 1.6.0). Data from the 10 experimental blocks were concatenated and down-sampled to 500Hz. Down-sampled data were then high-pass filtered at 1 Hz (using a 4th order Butterworth filter, effective order 8) and notch filtered around 50Hz and harmonics (using an 8th order Butterworth filter, effective order 16). Subsequently, data were epoched in a window between 300ms before and 1000ms after stimulus onset. Invalid trials (i.e. aborts of the laser) were removed, followed by a manual removal of extremely noisy epochs as determined by visual inspection. Data was further low-pass filtered with a cutoff frequency of 30Hz (4th order Butterworth filter) and either re-referenced to the average of all EEG electrodes (for analysis of the N2P2 complex) or to Fz (for analysis of the N1).

Data analysis – ESG. All analyses were performed using Python 3.10 and MNE (https://mne.tools/stable/index.html; version 1.6.0). First, in order to remove possible artefacts resulting from stimulation, data were linearly interpolated between -13ms and

13ms relative to stimulus onset. Data were then down sampled to 1kHz, and notch filtered to remove powerline noise at 50Hz and all harmonics up to 200Hz with an IIR filter. Next, the cardiac artefact was removed using signal space projection with 6 projectors and the data were bandpass filtered from 30Hz to 150Hz using a 4th order Butterworth, zero-phase filter. The data were then epoched from –100ms to 300ms relative to stimulation, with the baseline period defined from –100ms to –10ms. Finally, CCA was applied as described previously for Experiment 1 and Experiment 2, with the onset and duration of the training window changed based on the following reasoning.

Surface recordings show that with appropriate task / analysis, one can observe laser-evoked cortical responses peaking as soon as 83ms (EEG data; Valeriani et al., 2000) or 98ms (MEG-data; onset at 84ms; Wang et al., 2007) after stimulation onset, with invasive recordings revealing that the onset of cortical responses to laser stimulation can be early as ~70ms in S1 (Ohara et al., 2004). Invasive thalamic recordings demonstrate spikes between 60 and 70ms (Kobayashi et al., 2009) and induced responses in the gamma range at ~90ms (Kim et al., 2015) after laser stimulation (but see Bastuji et al. (2016) for later responses). fMRI-EEG fusion results points towards thalamic responses to laser stimulation from 65ms (in a lateral nucleus) and 89ms (in a medial nucleus) onwards (Tu et al., 2024). Together, these evoked-response data suggest that initial spinal responses could occur even before 60ms. With respect to conduction-velocity data, estimates of human spinothalamic tract conduction velocity vary between laboratories and employed methods (see Iannetti et al., 2001 and responses by Rossi et al. as well as Kagiki) and have been shown to differ in various spinothalamic pathways (Tsuji et al. 2006; Valeriani et al. 2007; see also Treede & Lenz, 2006). We thus did not base our estimation as to when expect spinal responses on these estimates, but instead relied on peripheral nerve conduction velocity estimates of A-delta fibres mediating responses to laser stimulation. These have been estimated to vary between 9-18m/s (Kakigi et al., 1991: 9m/s; Tran et al., 2001: 11m/s; Obi et al., 2007: 13m/s; Bromm & Treede, 1987: 16m/s; Kobayashi et al., 2009: 18m/s) and thus suggest possible initial spinal responses to occur roughly 45ms to 90ms after stimulation when i) considering an approximate distance of 80cm between hand dorsum and spinal cord and ii) ignoring any delay between laser stimulation onset and action potential generation in the peripheral nerve. Based on the above considerations, we trained CCA on a time-window of 45ms to 90ms after laser stimulation onset - this is only a heuristic for this first proof-of-principle experiment and it is likely that future studies investigating electrophysiological spinal responses in much more detail might lead to more optimized training windows.

For each participant, the first CCA component (as ranked by their canonical correlation coefficient) was selected and the resulting time-courses were averaged across participants to obtain a group-average response. Since CCA is not sensitive to the polarity of the signal and since we observed a negative deflection at ~50ms in the component time-course in 6 out of 7 participants (but a positive deflection at this time-point in participant 2), we multiplied this participant's time-course by -1 and used this sign-inverted time-course in all further analyses (similar to the procedure used in Experiment 1 and Experiment 2). In order to demonstrate the robustness of the obtained results, we also performed a within-participant four-fold split of the data (i.e. first split: trials 1,5,9,…; second split: trials 2,6,10,…; third split: trials 3,7,11,…; fourth split(trials: 4,8,12,…) after having applied the spatial filter and then averaged the results of each fold across participants."

Figure 8:

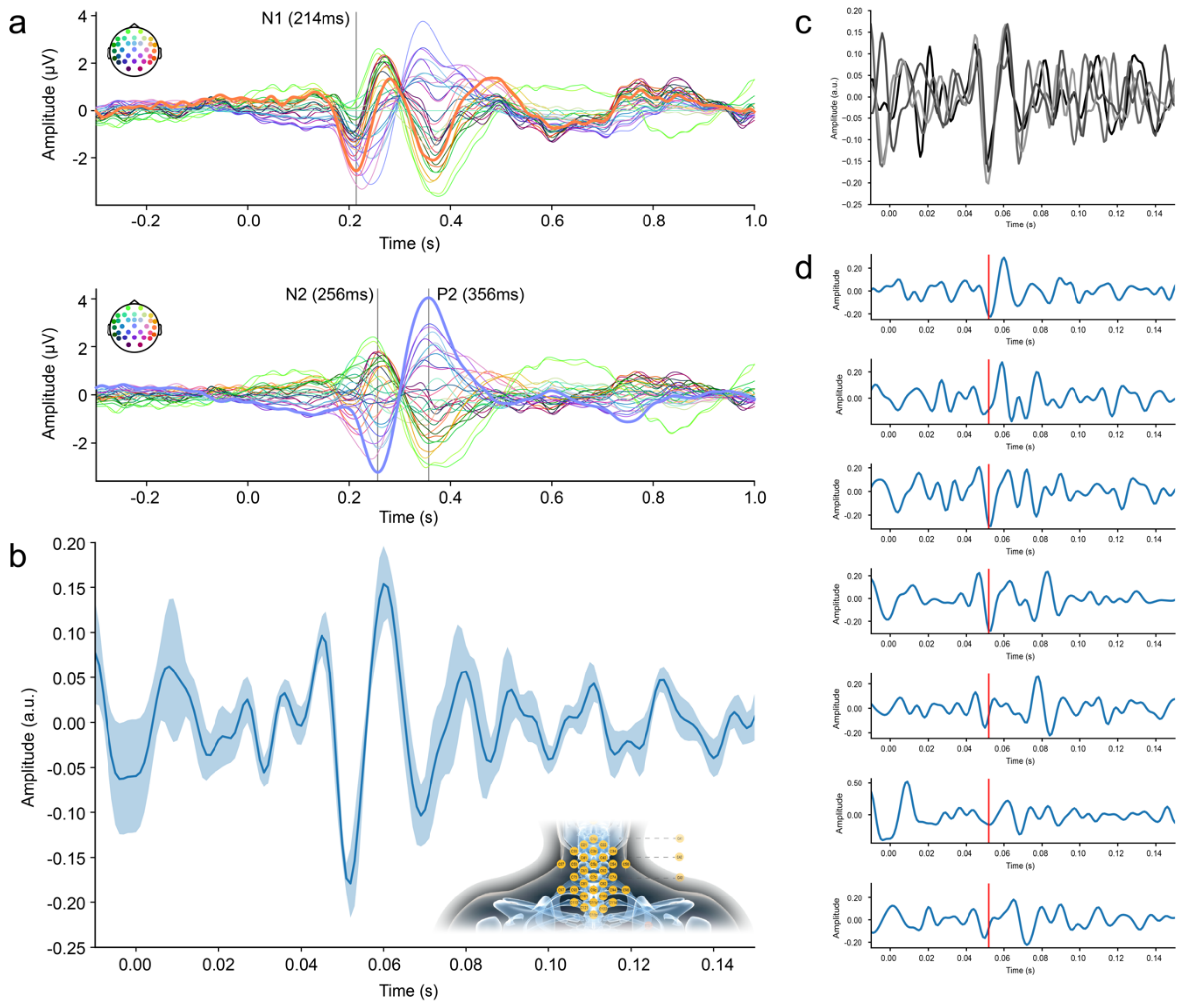

Figure legend: Cortical and spinal LEPs. a) Grand-average (N = 7) cortical LEPs, obtained from single electrodes and consisting of N1 (latency: 214ms; data referenced to Cz) in the top row and N2P2 (latency: 256ms and 356ms; data referenced to average) in the bottom row; line colors correspond to the electrodes depicted schematically the top left of each panel, with thick lines corresponding to electrodes of interest (N1: T8, N2P2: Cz). b) Grand-average spinal LEPs, obtained from CCA and showing the most prominent deflection at 52ms. The thick line corresponds to the group-average of the first CCA component and the shaded band to the standard error of the mean across participants (amplitudes are in arbitrary units). The electrode patch of Experiment 3 is shown as an inset. c) A four-fold split of trials (after having applied the spatial filter) shows a consistent response in each split at the previously shown latency (amplitudes are in arbitrary units and the grey lines reflect different splits). d) Single-participant (participants ordered from top (1) to bottom (7)) time-courses of the first CCA component, with the time-course of participant 2 having been multiplied by -1 (due to the polarity insensitivity of CCA); amplitudes are in arbitrary units. The red line indicates the time-point of maximal deflection observed in the group-level data.

It is able to provide results which are anatomically plausible etc. This kind of analysis would make more convincing the overall translational possibility of this approach.

We thank the reviewer for bringing up the important point of anatomical plausibility. Since the description of our already existing data in the Results and Discussion section were likely not detailed enough, we have now improved it even further.

Results (L212-218): "… our multi-channel set-up allowed for the first time to estimate the potentials' spatial topography (Figure 3B and 3F), which showed a radial dipole at peak latency, with a centre over the spinal cord, close to the spinal segments targeted by the electrical stimulation at wrist and ankle. Importantly, the topographies show that N13 and N22 responses are consistently limited to the relevant electrode-grid (cervical for upper-limb and lumbar for lower limb stimulation), with no evidence for responses in the irrelevant electrode grid, thus presenting a spatial double-dissociation.".

Discussion (L489-499): "… we made use of our multi-channel setup to investigate the spatial distribution of cervical and lumbar SEPs: both presented as radial dipoles, slightly above the spinous process of vertebra C6 for the N13 and slightly below the spinous process of vertebra L1 for the N22 and with a sagittal centre over the spinal cord, speaking against a myogenic origin (which would be expected to result in a more lateralized distribution; Jiang et al., 2021). Importantly, our results show a high degree of anatomical plausibility, not only by being centred close to the spinal segment of interest, but also by showing a spatial double-dissociation: the upper-limb N13 is clearly localized in cervical but not lumbar areas and the lower-limb N22 is clearly localized in lumbar but not cervical areas. To our knowledge, such a spatial characterization of spinal SEPs is unique, as even modern MSG-studies are limited to much smaller spatial windows (Akaza et al., 2021; Ushio et al., 2019; for ESG, see Desmedt & Huy, 1984) and would thus not allow for such insights".

Referenced literature

Akaza, M., Kawabata, S., Ozaki, I., Miyano, Y., Watanabe, T., Adachi, Y., Sekihara, K., Sumi, Y., Yokota, T., 2021. Noninvasive measurement of sensory action currents in the cervical cord by magnetospinography. Clin Neurophysiol 132, 382–391. https://doi.org/10.1016/j.clinph.2020.11.029

Basbaum, A.I., Bautista, D.M., Scherrer, G., Julius, D., 2009. Cellular and molecular mechanisms of pain. Cell 139, 267–284. https://doi.org/10.1016/j.cell.2009.09.028

Bastuji, H., Frot, M., Mazza, S., Perchet, C., Magnin, M., Garcia-Larrea, L., 2016. Thalamic Responses to Nociceptive-Specific Input in Humans: Functional Dichotomies and Thalamo-Cortical Connectivity. Cereb Cortex 26, 2663–2676. https://doi.org/10.1093/cercor/bhv106

Blankertz, B., Dornhege, G., Krauledat, M., Müller, K.-R., Curio, G., 2007. The non-invasive Berlin Brain-Computer Interface: fast acquisition of effective performance in untrained subjects. Neuroimage 37, 539–550. https://doi.org/10.1016/j.neuroimage.2007.01.051

Bromm, B., Treede, R.D., 1987. Pain Related Cerebral Potentials: Late and Ultralate Components. International Journal of Neuroscience 33, 15–23. https://doi.org/10.3109/00207458708985926

Corniani, G., Saal, H.P., 2020. Tactile innervation densities across the whole body. Journal of Neurophysiology 124, 1229–1240. https://doi.org/10.1152/jn.00313.2020

De Clercq, W., Vergult, A., Vanrumste, B., Van Paesschen, W., Van Huffel, S., 2006. Canonical correlation analysis applied to remove muscle artifacts from the electroencephalogram. IEEE Trans Biomed Eng 53, 2583–2587. https://doi.org/10.1109/TBME.2006.879459

Desmedt JE, Huy NT. BIT-mapped colour imaging of the potential fields of propagated and segmental subcortical components of somatosensory evoked potentials in man. Electroencephalogr Clin Neurophysiol. 1984;58: 481–497. https://doi.org/10.1016/0013-4694(84)90037-3

Fedele, T., Scheer, H.-J., Burghoff, M., Waterstraat, G., Nikulin, V.V., Curio, G., 2013. Distinction between added-energy and phase-resetting mechanisms in non-invasively detected somatosensory evoked responses. Annu Int Conf IEEE Eng Med Biol Soc 2013, 1688–1691. https://doi.org/10.1109/EMBC.2013.6609843

Haufe, S., Meinecke, F., Görgen, K., Dähne, S., Haynes, J.-D., Blankertz, B., Bießmann, F., 2014. On the interpretation of weight vectors of linear models in multivariate neuroimaging. Neuroimage 87, 96–110. https://doi.org/10.1016/j.neuroimage.2013.10.067

Heinricher, M.M., Tavares, I., Leith, J.L., Lumb, B.M., 2009. Descending control of nociception: Specificity, recruitment and plasticity. Brain Res Rev 60, 214–225. https://doi.org/10.1016/j.brainresrev.2008.12.009

Helmer, M., Warrington, S., Mohammadi-Nejad, A.-R., Ji, J.L., Howell, A., Rosand, B., Anticevic, A., Sotiropoulos, S.N., Murray, J.D., 2024. On the stability of canonical correlation analysis and partial least squares with application to brain-behavior associations. Commun Biol 7, 217. https://doi.org/10.1038/s42003-024-05869-4

Iannetti, G.D., Cruccu, G., Manfredi, M., 2001. The problem of conduction velocity of the human spinothalamic tract. Clin Neurophysiol 112, 1113–1116. https://doi.org/10.1016/s1388-2457(01)00493-x

Iannetti, G.D., Baumgärtner, U., Tracey, I., Treede, R.D., Magerl, W., 2013. Pinprick-evoked brain potentials: a novel tool to assess central sensitization of nociceptive pathways in humans. J Neurophysiol 110, 1107–1116. https://doi.org/10.1152/jn.00774.2012

Jiang, N., Wang, L., Huang, Z., Li, G., 2021. Mapping Responses of Lumbar Paravertebral Muscles to Single-Pulse Cortical TMS Using High-Density Surface Electromyography. IEEE Trans Neural Syst Rehabil Eng 29, 831–840. https://doi.org/10.1109/TNSRE.2021.3076095

Kakigi, R., Endo, C., Neshige, R., Kuroda, Y., Shibasaki, H., 1991. Estimation of conduction velocity of A delta fibers in humans. Muscle Nerve 14, 1193–1196. https://doi.org/10.1002/mus.880141209

Kim, J.H., Chien, J.H., Liu, C.C., Lenz, F.A., 2015. Painful cutaneous laser stimuli induce event-related gamma-band activity in the lateral thalamus of humans. J Neurophysiol 113, 1564–1573. https://doi.org/10.1152/jn.00778.2014

Kobayashi, K., Winberry, J., Liu, C.C., Treede, R.D., Lenz, F.A., 2009. A painful cutaneous laser stimulus evokes responses from single neurons in the human thalamic principal somatic sensory nucleus ventral caudal (Vc). J Neurophysiol 101, 2210–2217. https://doi.org/10.1152/jn.91347.2008

Kuner, R., 2015. Spinal excitatory mechanisms of pathological pain. Pain 156 Suppl 1, S11-17. https://doi.org/10.1097/j.pain.0000000000000118

Lejeune, N., Petrossova, E., Frahm, K.S., Mouraux, A., 2023. High-speed heating of the skin using a contact thermode elicits brain responses comparable to CO2 laser-evoked potentials. Clin Neurophysiol 146, 1–9. https://doi.org/10.1016/j.clinph.2022.11.008

Mandeville, G.K., Roscoe, J.T., 1971. Fundamental Research Statistics for the Behavioral Sciences.

Michel, C.M., Murray, M.M., 2012. Towards the utilization of EEG as a brain imaging tool. Neuroimage 61, 371–385. https://doi.org/10.1016/j.neuroimage.2011.12.039

Obi, T., Takatsu, M., Yamazaki, K., Kuroda, R., Terada, T., Mizoguchi, K., 2007. Conduction velocities of Adelta-fibers and C-fibers in human peripheral nerves and spinal cord after CO2 laser stimulation. J Clin Neurophysiol 24, 294–297. https://doi.org/10.1097/WNP.0b013e318038f45f

Ohara, S., Crone, N.E., Weiss, N., Treede, R.-D., Lenz, F.A., 2004. Cutaneous painful laser stimuli evoke responses recorded directly from primary somatosensory cortex in awake humans. J Neurophysiol 91, 2734–2746. https://doi.org/10.1152/jn.00912.2003

Plaghki, L., Mouraux, A., 2005. EEG and laser stimulation as tools for pain research. Curr Opin Investig Drugs 6, 58–64.

Pratt, H., Starr, A., Amlie, R.N., Politoske, D., 1979. Mechanically and electrically evoked somatosensory potentials in normal humans. Neurology 29, 1236–1244. https://doi.org/10.1212/wnl.29.9_part_1.1236

Pratt, H., Starr, A., 1981. Mechanically and electrically evoked somatosensory potentials in human: scalp and neck distributions of short latency components. Electroencephalogr Clin Neurophysiol 51, 138–147. https://doi.org/10.1016/0013-4694(81)90002-x

Reni, L., Ratto, S., Abbruzzese, G., Abbruzzese, M., Favale, E., 1988. Do muscle afferents contribute to the cervical response evoked by electrical stimulation of the median nerve in man? Exp Brain Res 72, 305–315. https://doi.org/10.1007/BF00250253

Spüler, M., Walter, A., Rosenstiel, W., Bogdan, M., 2014. Spatial filtering based on canonical correlation analysis for classification of evoked or event-related potentials in EEG data. IEEE Trans Neural Syst Rehabil Eng 22, 1097–1103. https://doi.org/10.1109/TNSRE.2013.2290870

Stephani, T., Hodapp, A., Jamshidi Idaji, M., Villringer, A., Nikulin, V.V., 2021. Neural excitability and sensory input determine intensity perception with opposing directions in initial cortical responses. Elife 10, e67838. https://doi.org/10.7554/eLife.67838

Tarkka, I.M., Teräväinen, H.T., 1985. Short latency somatosensory evoked potentials during active hand movements. Am J Phys Med 64, 10–16.

Tran, T.D., Lam, K., Hoshiyama, M., Kakigi, R., 2001. A new method for measuring the conduction velocities of Abeta-, Adelta- and C-fibers following electric and CO(2) laser stimulation in humans. Neurosci Lett 301, 187–190. https://doi.org/10.1016/s0304-3940(01)01639-1

Treede, R.-D., Lenz, F.A., 2006. Passing lanes and slow lanes into the nociceptive network of the human brain. Pain 123, 223–225. https://doi.org/10.1016/j.pain.2006.05.014

Tsuji, T., Inui, K., Kojima, S., Kakigi, R., 2006. Multiple pathways for noxious information in the human spinal cord. Pain 123, 322–331. https://doi.org/10.1016/j.pain.2006.03.009

Tu, Y., Li, Z., Zhang, L., Zhang, H., Bi, Y., Yue, L., Hu, L., 2024. Pain-preferential thalamocortical neural dynamics across species. Nat Hum Behav 8, 149–163. https://doi.org/10.1038/s41562-023-01714-6

Ushio, S., Hoshino, Y., Kawabata, S., Adachi, Y., Sekihara, K., Sumiya, S., Ukegawa, D., Sakaki, K., Watanabe, T., Hasegawa, Y., Okawa, A., 2019. Visualization of the electrical activity of the cauda equina using a magnetospinography system in healthy subjects. Clin Neurophysiol 130, 1–11. https://doi.org/10.1016/j.clinph.2018.11.001

Valeriani, M., Le Pera, D., Restuccia, D., de Armas, L., Miliucci, R., Betti, V., Vigevano, F., Tonali, P., 2007. Parallel spinal pathways generate the middle-latency N1 and the late P2 components of the laser evoked potentials. Clin Neurophysiol 118, 1097–1104. https://doi.org/10.1016/j.clinph.2007.01.015

Valeriani, M., Restuccia, D., Le Pera, D., Fiaschetti, L., Tonali, P., Arendt-Nielsen, L., 2000. Unmasking of an early laser evoked potential by a point localization task. Clin Neurophysiol 111, 1927–1933. https://doi.org/10.1016/s1388-2457(00)00439-9

Wang, X., Inui, K., Kakigi, R., 2007. Early cortical activities evoked by noxious stimulation in humans. Exp Brain Res 180, 481–489. https://doi.org/10.1007/s00221-007-0878-3

Waterstraat, G., Fedele, T., Burghoff, M., Scheer, H.-J., Curio, G., 2015. Recording human cortical population spikes non-invasively--An EEG tutorial. J Neurosci Methods 250, 74–84. https://doi.org/10.1016/j.jneumeth.2014.08.013
